# Modeling Electrical Percolation to optimize the Electromechanical Properties of CNT/Polymer Composites in Highly Stretchable Fiber Strain Sensors

**DOI:** 10.1038/s41598-019-56940-8

**Published:** 2019-12-30

**Authors:** Sungmin Jung, Hyung Woo Choi, Felix Cosmin Mocanu, Dong-Wook Shin, Mohamed Foysol Chowdhury, Soo Deok Han, Yo-Han Suh, Yuljae Cho, Hanleem Lee, Xiangbing Fan, Sang Yun Bang, Shijie Zhan, Jiajie Yang, Bo Hou, Young Tea Chun, Sanghyo Lee, Luigi Giuseppe Occhipinti, Jong Min Kim

**Affiliations:** Electrical Engineering Division, Department of Engineering, University of Cambridge, 9 JJ Thomson Avenue, Cambridge, CB3 0FA United Kingdom

**Keywords:** Computational methods, Carbon nanotubes and fullerenes

## Abstract

A simulation model of electrical percolation through a three-dimensional network of curved CNTs is developed in order to analyze the electromechanical properties of a highly stretchable fiber strain sensor made of a CNT/polymer composite. Rigid-body movement of the curved CNTs within the polymer matrix is described analytically. Random arrangements of CNTs within the composite are generated by a Monte-Carlo simulation method and a union-find algorithm is utilized to investigate the network percolation. Consequently, the strain-induced resistance change curves are obtained in a wide strain range of the composite. In order to compare our model with experimental results, two CNT/polymer composite fibers were fabricated and tested as strain sensors. Their effective CNT volume fractions are estimated by comparing the experimental data with our simulation model. The results confirm that the proposed simulation model reproduces well the experimental data and is useful for predicting and optimizing the electromechanical characteristics of highly stretchable fiber strain sensors based on CNT/polymer composites.

## Introduction

A smart textile technology has been spotlighted due to their tremendous possibilities in wearable electronics application including sensors, displays and computing devices^1,2^. Owing to the conformability of textiles, fiber sensors embedded in textiles have been developed as communication interfaces between the human and its environment. In particular, a fiber strain sensor device knitted or woven in a smart textile is a key component to respond to motions of the human body^3^. In order to have a practical application of the fiber strain sensor in conjunction with the smart textile, it is required to operate in a wide range of the strain with high strain sensitivity^4,5^.

One of the promising materials for fiber strain sensors satisfying high stretchability and high sensitivity is the carbon nanotube (CNT) based polymer composite^6^. The electrical conduction of a polymer filled with 1-dimensional conductive structures, such as nanowires and CNTs, can be explained by formation of a percolation network based on conduction through CNTs and tunneling among neighboring CNTs^7^. Owing to the superior electrical and mechanical properties, CNT/polymer composite can give sufficient strain sensitivity in a wide operation range of strain just with a small amount of CNT filler^8^.

The electrical characteristics of CNT/polymer composites modified by different types of CNTs in various polymer types under a given strain have been of great interest to obtain an optimal design for the applications of fiber strain sensors^9^. However, evaluating the electrical conductivity of CNT/polymer composites through experimental trials towards development of behavioral models for design use is an expensive and inefficient method. It is certainly more efficient and economical to perform computational evaluations by using a mathematical model of the percolation physics governing the electrical charge transport in CNT/polymer composites.

The current literatures have focused on the modeling and simulation of the electrically conductive percolation network within CNT/polymer composites^10–15^. Here, a three-dimensional model based on the Monte-Carlo method has been proposed to describe the random distribution of CNTs in polymers^16^. These papers have investigated the change in electrical conductivity not only in relation to the variation of material parameters of CNTs and various polymers, but also, in terms of the mechanical strain induced by stretching CNT/polymer composites. However, they used a simulation model of a rigid-body movement of straight-lined CNTs under the strain by stretching, even though the shape of CNTs in a polymer network is considered to be in the form of random curves due to their high aspect ratio of CNTs^14^. Various attempts to develop physical models have been published^17–25^, with limited capability to accurately reproduce the behavior of CNT/polymer composites under large strain conditions. In the previous simulation model, the strain is limited to the range up to 10% where the linear approximation can be applied^18,22,26^. This model is not applicable to simulate fiber strain sensor operating in a wide range of strain. Therefore, a physical model applicable to a wide strain range with curved CNTs needs to be developed in order to investigate rigorously the resistance changes of CNT/polymer composites in highly responsive textile strain sensor applications.

In this work, the electrical conduction of a CNT/polymer composite under uniaxial stress is studied through the three-dimensional simulation of the electrical percolation of CNTs. The Monte-Carlo simulation method and a union-find algorithm are used to configure random distribution of CNTs and to investigate the formation of percolation pathway. A mathematical model for the rigid-body movement of curved CNTs is also developed for large strain of the highly stretchable fiber strain sensor. Based on our simulation model of electrical percolation for curved CNTs for a large strain of a composite, electrical properties of the composite are obtained for different lengths and the different curvature of CNTs. Finally, in order to compare our simulation results with the experiments, the fiber strain sensors are fabricated from CNT/polymer composites and their electromechanical properties are analyzed by our simulation model.

## Method

**Figure 1:**
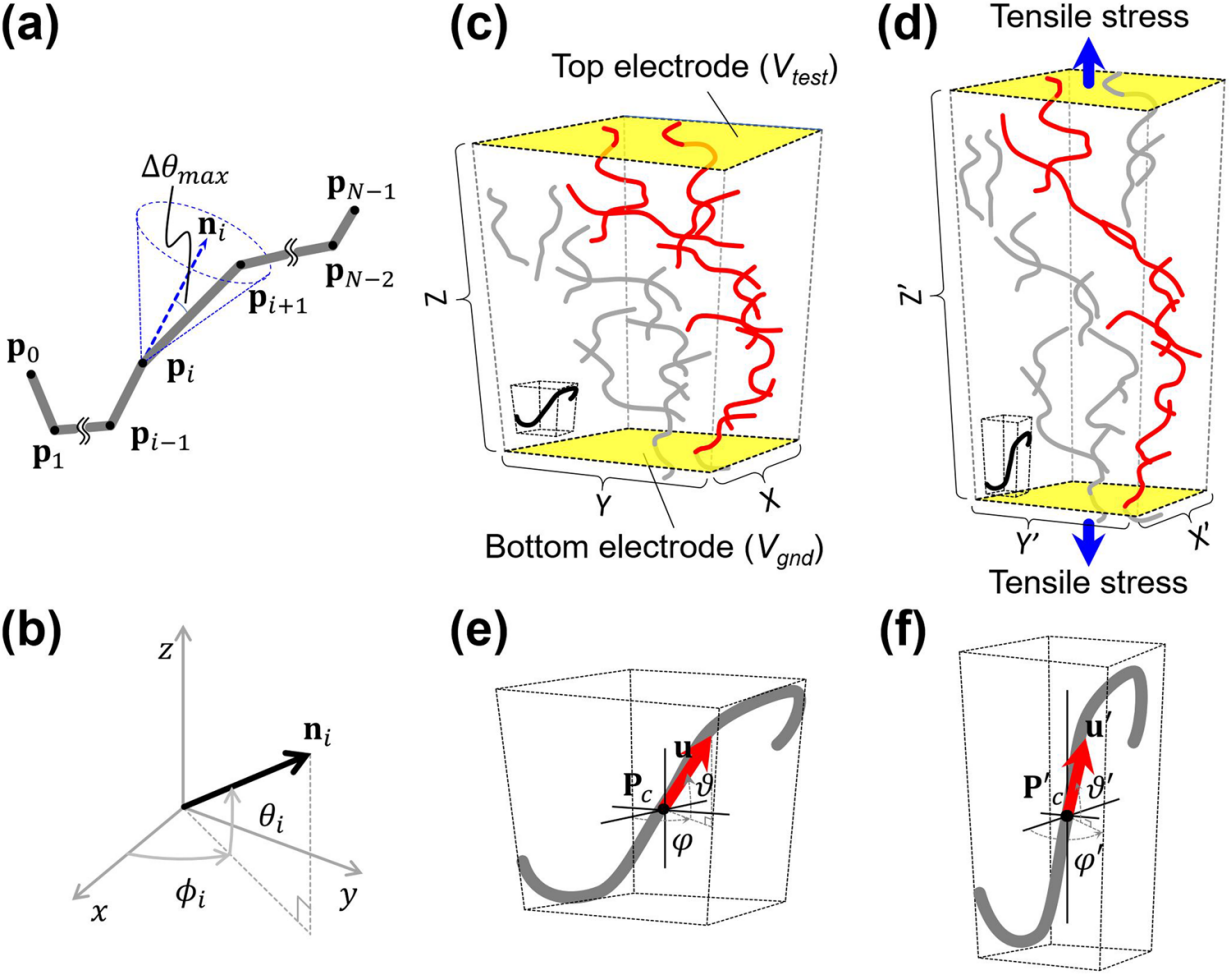
(Occhipinti) Configuration of CNTs in the polymer composite under strain by stretching. (**a**) Graphical representation of curved CNTs with a set of segmented lines, (**b**) definition of angles for each directional vector of segmented line. (**c**) Calculation domain without strain. Red lines indicate the CNTs in the percolation network. (**d**) Calculation domain with strain. Curved CNT (**e**) of initial configuration and (**f**) after the strain is given to the composite box.

To obtain the electrical conductivity of a CNT/polymer composite in a given strain condition, the geometrical configuration of the calculation domain is described in Fig. 1. In this work, the CNTs are approximated as a set of straight-line segments as shown in Fig. 1(a) in order to describe the curved shape of the CNTs^12–14^. Here, the vectors of each segmented line of the CNTs and their corresponding angles are defined in Fig. 1(b). Initially, the curved CNTs are distributed randomly within a composite box forming an electrical percolation network represented by the red lines between the top and bottom electrodes, as shown in Fig. 1(c). After a tensile stress is given to the composite in the *z*-direction, then the composite box is enlarged in *z*-direction and contracted in *x*- and *y*-directions as shown in Fig. 1(d). By deforming the composite box, the percolation network changes, and its corresponding electrical conductance is also changed. Individual CNTs as in Fig. 1(e) are translated and reoriented by the strain of the stretched composite box as shown in Fig. 1(f).

In order to configure CNTs in the calculation domain, the locations of all the CNTs are determined by a random process obtained from Monte-Carlo analysis. Then, the maximum deviation angle Δ*θ*_*max*_ shown in Fig. 1(a) is introduced to constrain the random curvature of CNTs. After single segmented line is generated randomly as a part of curved CNTs, the next adjacent segmented line is also randomly generated satisfying the angular constraint of **n**_*i*_∙**n**_*i*+1_ ≤ cosΔ*θ*_*max*_ where **n**_*i*_ and **n**_*i*+1_ are unit vectors of *i*-th and (*i* + 1)-th segments.

In order to predict the changes in electrical properties of the highly stretchable fiber strain sensor, the next step is to build a simulation model for curved CNTs under a large unidirectional strain of CNT/polymer composite. Assuming that the tensile stress is given in *z*-direction, the height of the composite box *Z* will be changed to be *Z*′ = (1 + *ε*)∙*Z* for the strain along the direction of tensile strain, *ε*. When the large strain is introduced, the length and width of the composite box *X* and *Y* in the transverse direction of the tensile stress are changed nonlinearly to *X*′ = (1 + *ε*)^−ν^ ∙*X* and *Y*′ = (1 + *ε*)^−ν^∙*Y* for the Poisson’s ratio *ν* of a given polymer.

Since the CNTs in a polymer material can be assumed to be a rigid body in a continuum, the CNTs should be translated and reoriented by the deformation of a given continuum through the model of rigid-body movement^26,27^. As CNTs are considered to be a curved line, which is a set of multiple straight-lines connected together in this work, additional effort is needed for the rigid-body movement model to describe the translation and reorientation of a curved CNTs with vector representation. Namely, to present mathematical forms of the translation and the reorientation of CNTs, we define the center point **p**_*c*_ along the curved line of CNT and the vector **u** representing the average direction of CNTs. The center point of the CNTs can then be translated by the transformation relationship of **p**′_*c*_ = (*x*′_*c*_, *y*′_*c*_, *z*′_*c*_)^T^ = [(1 + *ε*)^−ν^∙*x*_*c*_, (1 + *ε*)^−ν^∙*y*_*c*_, (1 + *ε*)∙*z*_*c*_]^T^ from the coordinate transformation between the strained and unstrained coordinates. Here, the upper script T is the transpose operation of a given vector.

The directional vector representing the average direction, **u**, in Fig. 1(e) can in turn be expressed by the following equation.

1 $${\bf{u}}=\frac{1}{{l}_{cnt}}\mathop{\sum }\limits_{i=1}^{N-1}({{\bf{p}}}_{i}-{{\bf{p}}}_{i-1})={({u}_{x},{u}_{y},{u}_{z})}^{T}={(\cos \vartheta \cos \varphi ,\cos \vartheta \sin \varphi ,\cos \vartheta )}^{T},$$

where **p**_*i*_ is the location of the *i*-th nodal point and *N* is the total number of nodes on the CNTs. In the same manner, the directional vector representing the average direction of CNTs, **u**′ in Fig. 1(f), after the strain is applied, can be expressed as **u**′ = (*u*′_*x*_, *u*′_*y*_, *u*′_*z*_)^T^ = *u*′(cos*ϑ*′cos*φ*′, cos*ϑ*′sin*φ*′, sin*ϑ*′)^T^. Then, the relationships of tilt and azimuthal angles are obtained as described in the equations (2), by the transformation relationships between the original and deformed coordinates^27^.

2.a $$\vartheta ^{\prime} ={\tan }^{-1}[{(1+\varepsilon )}^{1-\nu }\cdot \,\tan \,\vartheta ],$$

2b $$\varphi ^{\prime} =\varphi .$$

Finally, all the nodal points **p**′_*i*_ = (*x*′_*i*_, *y*′_*i*_, *z*′_*i*_)^*T*^ on the CNTs can be translated and rotated by the matrix relationship of the following equation,

3 $${{\bf{p}}^{\prime} }_{i}={{\bf{R}}}_{d}\cdot ({{\bf{p}}}_{i}-{{\bf{p}}}_{c})+{{\bf{p}}^{\prime} }_{c},$$

where the rotation matrix by the deformation, **R**_*d*_, can be expressed as^28^

4 $${{\bf{R}}}_{d}(\varDelta \vartheta )=[\begin{array}{ccc}\cos \,{\Delta }\vartheta +{\sin }^{2}\varphi (1-\cos \,\,{\Delta }\vartheta ) & -\sin \,\varphi \,\cos \,\varphi (1-\,\cos \,{\Delta }\vartheta ) & -\cos \,\varphi \,\sin \,{\Delta }\vartheta \\ -\sin \,\varphi \,\cos \,\varphi (1-\,\cos \,{\Delta }\vartheta ) & \cos \,{\Delta }\vartheta +{\cos }^{2}\,\varphi (1-\,\cos \,{\Delta }\vartheta ) & -\sin \,\varphi \,\sin \,{\Delta }\vartheta \\ \cos \,\varphi \,\sin \,{\Delta }\vartheta  & \sin \,\varphi \,\sin \,{\Delta }\vartheta  & \cos \,{\Delta }\vartheta \end{array}].$$

Here, Δ*ϑ* is defined by Δ*ϑ* =  *ϑ*′−*ϑ* from the transformation between angles given in equations (2). All the CNTs are first relocated and reoriented according to the model of rigid-body movement for curved CNTs under the given strain. Then the simulation of electrical percolation is performed for the redistributed CNTs in the polymer structure.

**Figure 2:**
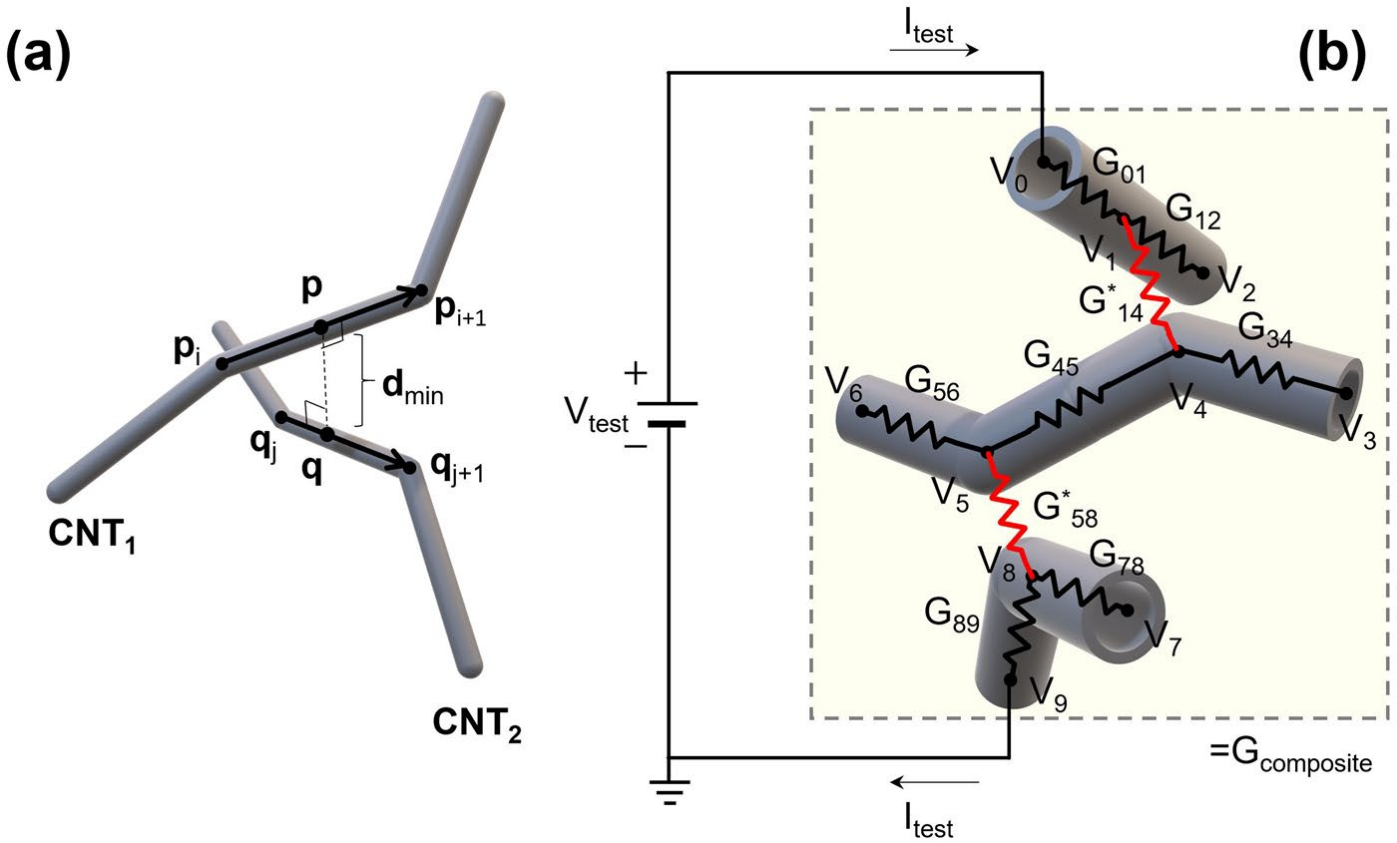
(Occhipinti) Geometrical and electrical relationships among the CNTs. (**a**) Minimum distance between two curved CNTs. (**b**) Schematic illustration of electrical network from the percolation network.

A critical step in the electrical percolation simulation is obtaining the set of CNTs that belongs to the percolation network. In this research, a union-find algorithm is applied to obtain the electrical percolation network from top to bottom electrodes through the entire CNT/polymer composite with a given distribution of the CNTs^29,30^. When two adjacent CNTs are closer than any given cutoff distance, they are combined together by the union-find algorithm. The graphical representation of minimum distance between two adjacent CNTs is shown in Fig. 2(a) while an example of the electrical network translated from a percolation network of a CNT/polymer composite is described in Fig. 2(b). Two conductance mechanisms, such as intrinsic conductance and by tunneling effect, are used to model the conducting paths in the electrical network. On one hand, the intrinsic conductance *G*_*ij*_ along the CNTs between two neighboring nodal points is determined by Eq. (5).

5 $${G}_{ij}={\sigma }_{cnt}\frac{\pi {r}_{cnt}^{2}}{{l}_{ij}}$$

Here, *σ*_*cnt*_ is a conductivity of the CNTs, *r*_*cnt*_ is the outermost radius of CNT and *l*_*ij*_ is the length between *i*-th and *j*-th nodal points on the CNTs, respectively. On the other hand, the tunneling conductance between two CNTs can be calculated by the Eq. (6) if when two CNTs are separated by a polymer with thickness *d*_*min*_ expressed in Fig. 2(a)^31^.

6 $${G}_{ij}^{\ast }=\{\begin{array}{cc}{G}_{0}M\,{\exp }\,(-\frac{4\pi {d}_{vdw}}{h}\sqrt{2{m}_{e}\Delta E}), & \,{d}_{min}\le {d}_{vdw}+({r}_{i}+{r}_{j})\\ {G}_{0}M\,{\exp }\,[-\frac{4\pi ({d}_{min}-{r}_{i}-{r}_{j})}{h}\sqrt{2{m}_{e}\Delta E}], & \,{d}_{min} > {d}_{vdw}+({r}_{i}+{r}_{j})\end{array}$$

where *i* and *j* are integers denoting the index of given nodal points on each CNT. The superscript * of the conductance is used to the tunneling conductance contribution. Here, the soft-core model allowing CNT bodies to cross each other is applied to determine an effective distance for calculating a tunneling probability between two CNTs^14,15^. *d*_*vdw*_ is the Van der Waals distance and *G*_0_ is a quantized conductance defined by *G*_0_ = 7.748×10^−5^ S. *M* is the number of conduction channels for the tunneling path. *m*_*e*_ is the electron mass and Δ*E* is defined as the work function difference between the polymer and the CNTs, respectively.

In the example of electrical configuration shown in Fig. 2(b) there are 8 unknown voltages from V_1_ to V_8_ formed in the circuit system. The matrix equation for the 8 unknown voltages can be derived by Kirchhoff’s current law. However, since there are huge number of unknown voltages given the large amount of CNTs in the calculation domain of a practical simulation, the linear system gives symmetric and sparse matrix with huge size. In this study, a conjugate gradient method is used for solving the huge matrix equation^32^. Finally, the equivalent conductance *G*_*composite*_ of a given electrical network is obtained by Ohm’s law from the calculated voltage distribution^14,33,34^.

## Results and Discussion

**Figure 3:**
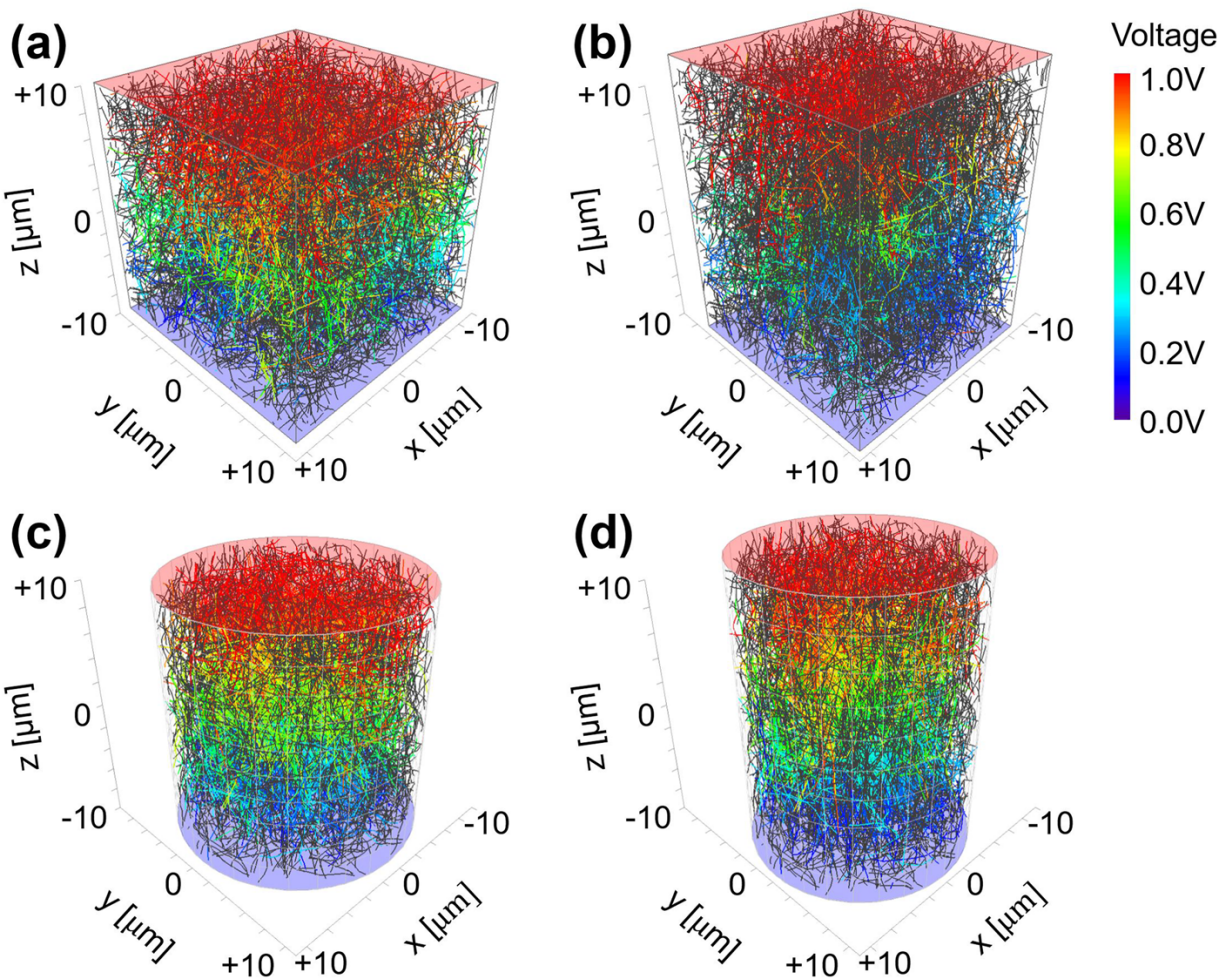
(Occhipinti) Graphical representation of CNTs in the calculation domain for the different number of CNTs. CNTs in the percolation network were plotted in rainbow color according to their simulated voltages. Simulated distributions of curved CNTs in a composite box under 0% and 20% strains are drawn in (**a**) and (**b**). The CNT distribution in a composite cylinder under 0% and 20% strains are also described in (**c**) and (**d**) for the simulation of fiber application as an example.

Figure 3 shows the three-dimensional distributions of CNTs and their electrical percolation network from our simulation model based on the Monte-Carlo method. Simulated distributions of curved CNTs in a composite box under 0% and 20% strains are drawn in Fig. 3(a,b). The CNT distribution in a composite cylinder under 0% and 20% strains are also reported in Fig. 3(c,d) for the simulation of the fiber application, as an example. The electrodes are illustrated as half transparent red and blue surfaces on the top and bottom faces of each composite structure. In Fig. 3, individual CNTs belonging to the percolation network are displayed by rainbow colors according to their voltage range from 1.0 V (red) to 0.0 V (blue) for voltages from top to bottom electrodes. The gray curves represent the CNTs, which are not forming part of the percolation network. The maximum deviation angle of the CNT curve Δ*θ*_*max*_ is limited to 30° representing the degree of CNT bending in our simulation model.

The size of the three-dimensional composite box is defined as *L*_*x*_ × *L*_*y*_ × *L*_*z*_ = 20 × 20 × 20 μm^3^ and the top and the bottom electrodes are located on the *z* = ±10 μm plane. The voltage difference between the top and the bottom electrodes was set to 1.0 V as a test voltage. CNTs are considered to have the average radius of 25 nm, with the average length of 5 μm and the conductivity was assumed to be 1.0 × 10^4^ S/m. As part of our study, a random number generator is used for the length and the radius of CNTs that follows Weibull and log-normal probability distribution functions, respectively^13,35^. The Van der Waals distance *d*_*vdw*_ and the cut-off distance *d*_*cutoff*_ between the outermost wall of two adjacent CNTs are considered to be 3.4 Å and 10.0 Å, respectively, in our simulation. The work function difference between the CNTs and the polymers Δ*E* is 1.0 eV and the number of conduction channels *M* is set at 400 for the calculation of conductance through electron tunneling paths.

One interesting finding is that the voltage distribution on the CNT belonging to the percolation network follows a nearly linear distribution from top to bottom as shown in Fig. 3. Since the uniformly distributed CNTs are connected to each other by electron tunneling, a linear distribution of voltage is likely to be formed throughout the entire region of CNT/polymer composite. Another important finding is that some of CNTs will become disconnected from the percolation network when the composite structure is strained, in line with our rigid-body movement model for curved CNTs as described in the previous section. These corresponding reduction of the number of CNTs connected to the percolation network leads to a reduction in overall electrical conductance. With the three-dimensional percolation network obtained in our simulation model, it is possible to analyse the electromechanical characteristics with respect to the CNT volume fraction in the composite, for the different parameters describing the shape of CNTs.

**Figure 4:**
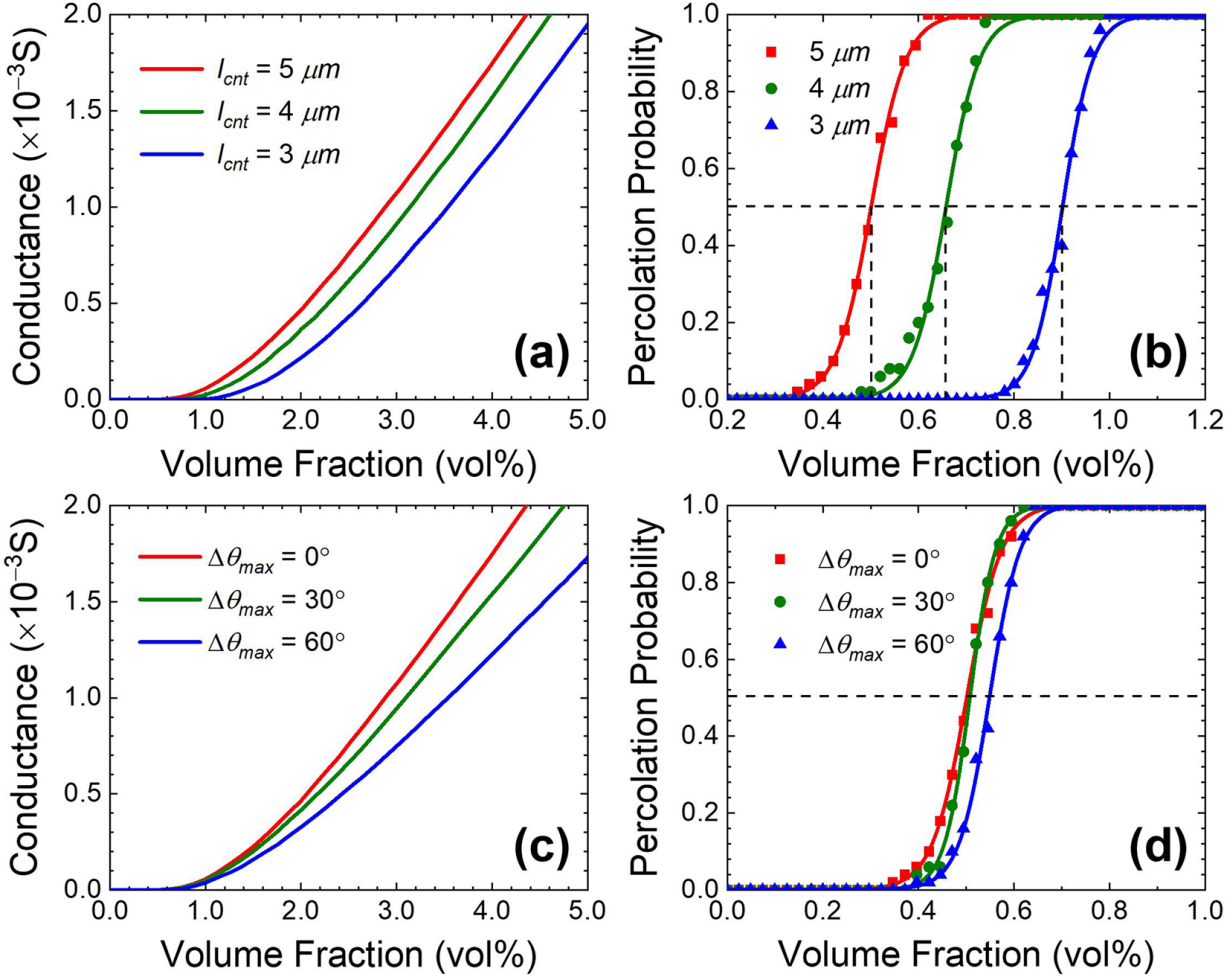
(Occhipinti) Electrical properties of CNT/polymer composite as a function of CNT volume fraction. (**a**) Conductance curves and (**b**) percolation probability for various lengths of CNTs and (**c**) conductance curves and (**d**) percolation probability for various maximum deviation angles of CNTs.

The simulated conductance and their percolation probability curves as a function of CNT volume fractions are plotted in Fig. 4. The conductance curves are plotted as solid, dashed and dotted lines while the simulated percolation probability data are also plotted as squares, circles and triangles for different CNT conditions such as their length (Fig. 4(a,b)) and maximum deviation angles (Fig. 4(c,d)), respectively. To maximize simulation reliability, percolation probability data are obtained from 50 simulations by configuring 50 random CNT distributions for a given calculation unit cell. The binomial logistic curves fitted to the percolation probability data are also drawn by red, green and blue lines for different CNT conditions in the percolation probability plots.

In Fig. 4(a,b), the conductance curves are obtained by the simulation for different values of the CNT length of 5 μm, 4 μm and 3 μm, respectively, and CNT diameter of 50 nm. From Fig. 4(a), it is shown that the volume fraction exhibiting percolation network formation of shorter CNT is larger than that of longer CNT. This is because the composite with shorter CNT length requires more CNTs to compensate its short length by increasing the number of tunneling paths in a percolation network. As shown in the figure, all the conductance curves and percolation probability curves with respect to CNT volume fractions agree well with previous reports^9,36,37^. By defining the critical volume fraction to be a volume fraction at the probability of 0.5 in Fig. 4(b), the critical volume fractions are obtained to be 0.5 vol%, 0.7 vol% and 0.9 vol% for the CNT lengths of 5 μm, 4 μm and 3 μm, respectively. The simulated conductances and their percolation probability characteristics for different maximum deviation angles of Δ*θ*_max_ = 0°, 30° and 60° are also obtained and plotted in Fig. 4(c,d). Here, the maximum deviation angle represents the curvature of CNTs, where CNTs are modeled as straight lines at Δ*θ*_max_ = 0° and curly one for larger Δ*θ*_max_. It is found that the slope of conductance increase becomes steeper as the maximum deviation angle of CNT decreases. It is also found that the critical volume fraction is affected significant changes for curlier CNTs from *Δθ*_*max*_ = 30° to 60°, as curly CNTs in the percolation network result in longer effective current paths through the composite. It is noted however that the percolation probability curves of *Δθ*_*max*_ = 0° and 30° show similar shapes due to the similarity of their critical volume fractions and resultant percolation networks.

The results of our simulation show that the degree of bending of CNTs has significant influence on the electrical properties, meaning that the model of rigid-body movement of curved CNTs is necessary in order to enable more rigorous analysis and effective simulation of CNT/polymer composites.

**Figure 5:**
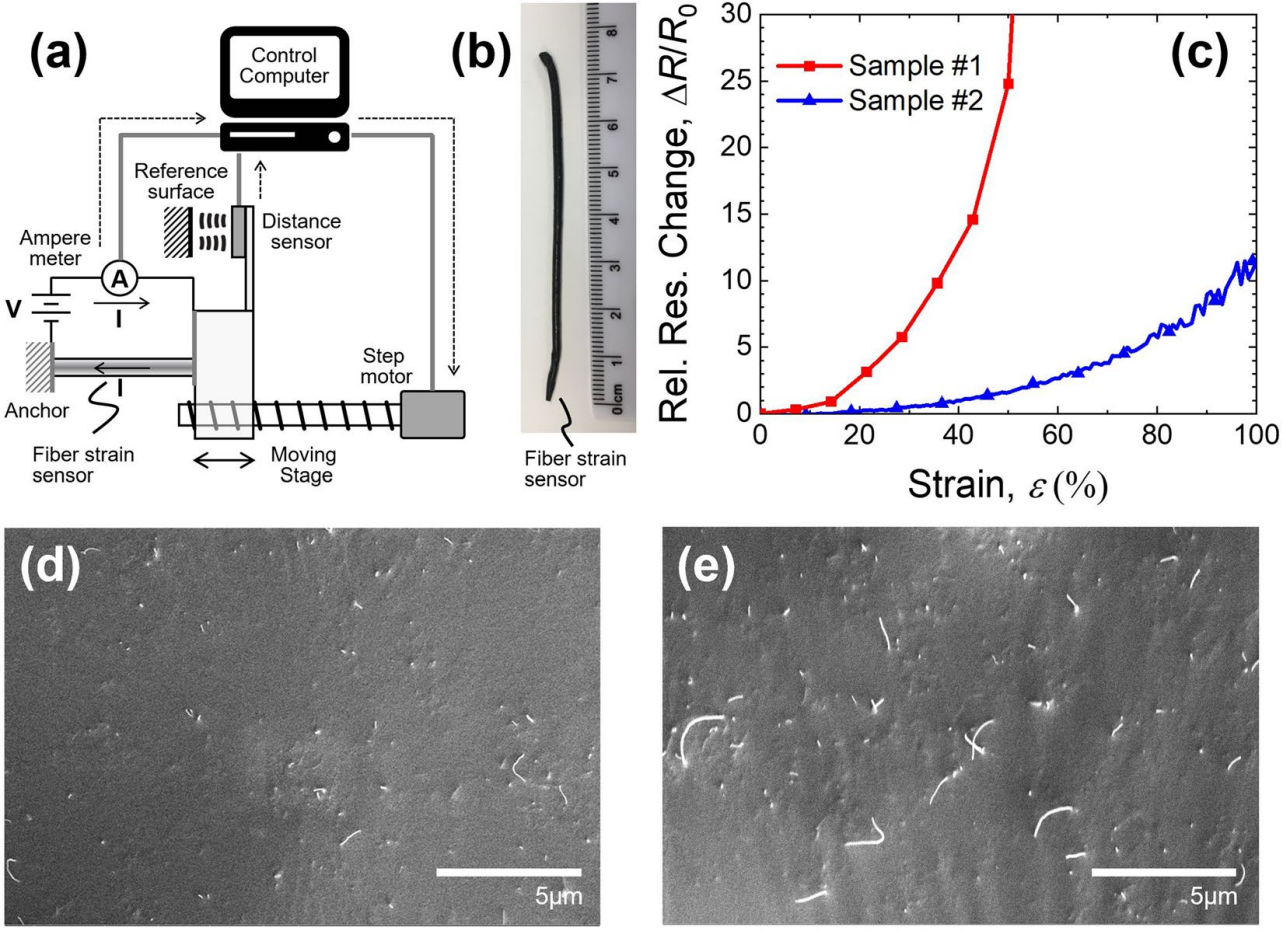
(Occhipinti) Experiments on the fiber strain sensor fabricated by the CNT/polymer composite. (**a**) Experimental setup, (**b**) a snapshot of fabricated fiber strain sensor, and (**c**) the relative resistance change curves for two different samples. Cross-sectional SEM images of CNT/polymer composite fiber strain sensors for (**d**) sample #1 and (**e**) sample #2.

To obtain experimental data of resistance changes in a CNT/polymer composite for a large range of strain, fiber strain sensors were fabricated from the CNT/polymer composite. An experimental setup to measure the resistance changes under strain conditions is schematically drawn in Fig. 5(a) and the fabricated fiber strain sensor made of a CNT/polymer composite shown in Fig. 5(b). The strain-induced resistance change in the samples was measured by the given experimental setup and shown in Fig. 5(c). Two samples of the fiber strain sensor possessing 3.2 vol% CNTs with the diameter in a range of 110–340 nm and the length in a range of 5–9 μm and Ecoflex (Pt-catalyzed silicon rubber) were used for the CNT/polymer composite fabrication. Two sample fibers are numbered to #1 and #2 in our experiments.

The stain-induced resistance change of fabricated fibers are plotted with solid lines of squares and triangles in Fig. 5(c). As shown in the figure, two fibers show quite different behaviors even though they were fabricated with the same CNT volume fraction of 3.2 vol%. As shown in Fig. 5(c), the fiber sensor #1 shows an abrupt increase in the resistance changes higher than 50% strain condition, while the fiber sensor #2 shows monotonical resistance increase over the entire strain range. The values of normalized resistance changes obtained are 25 at 50% strain for sample #1 and 10 at 100% strain for sample #2, respectively. From these results, it is thought that each sample has a different percolation network and that the percolation network of sample #2 is more densely interconnected than the one of sample #1. Since the percolation network is strongly affected by the volume fraction, significantly different resistance change stems from local variation of effective CNT volume fraction.

The cross-sectional images for each fibre strain sensor of sample #1 and #2 were taken by scanning electron microscopy (SEM) and presented in Fig. 5(d,e), in order to show the random network of CNTs in our CNT/polymer composites. From the cross-sectional SEM images of sample #1 and sample #2 in Fig. 5(d,e), inhomogeneous dispersion or difference of CNT density were observed, supporting the assumption of different percolation network or volume fractions between two samples. Since the difference of the conduction behavior for the strain may come from the shortening of CNTs by fabrication or inhomogeneous dispersion of CNTs, it is concluded that these factors can produce the different volume fractions observed between two samples. Finally, it is worth noting that, even though two samples show different slopes in resistance increase, both samples show the same trend of exponential increase with respect to an increased strain. From the results, it can be concluded that the curves of normalized resistance changes of CNT/polymer composites show an exponential behavior with respect to the strain. For that, it is assumed that the increment rate in the exponential function of the resistance change curve is determined by the connectivity of percolation network, which is affected by the effective CNT volume fraction in the composite.

**Figure 6:**
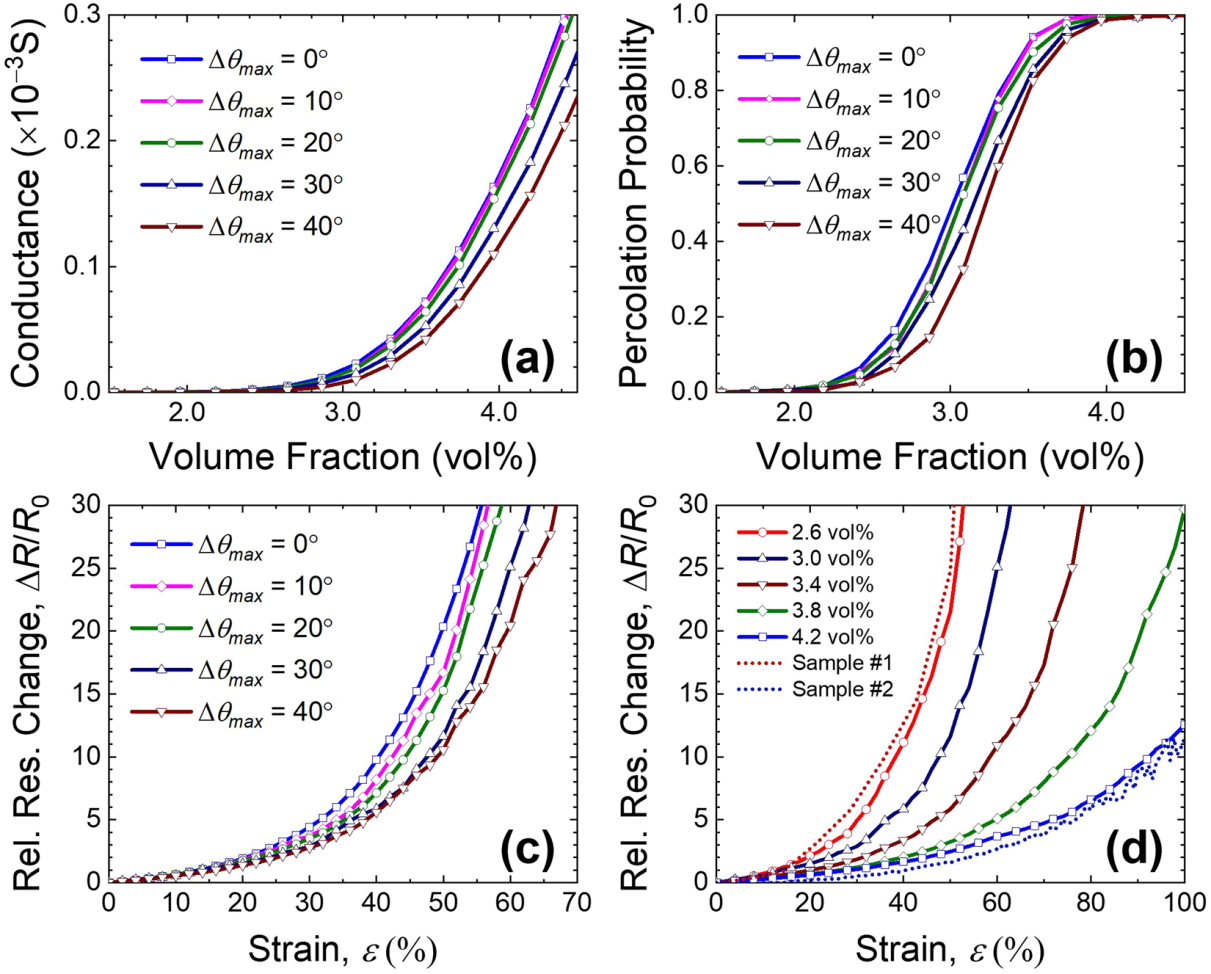
(Occhipinti) Simulated electrical and mechanical properties of CNT/polymer composite under different values of volume fraction and strain parameters. **(a**) Conductance curves and (**b**) percolation probability curves as a function of volume fraction for different values of the maximum deviation angles. The relative resistance change curve as a function of strain for (**c**) different maximum deviation angles and (**d**) different volume fraction around the critical volume fraction. The relative resistance change curves from experimental results of sample #1 and #2 are plotted together in (**d**).

In order to analyze further the experimental results given in Fig. 5, the resistance change curves are replicated using our three-dimensional simulation model for curved CNTs by a large strain. Figure 6 shows all the simulation results of electrical and mechanical properties of the CNT/polymer composite under the condition of our experiments with respect to the volume fraction of CNTs and the strain up to 100% of the fiber strain sensor. The average length and the radius of CNT in the simulation are 7 μm and 230 nm in order to model the CNTs used in our experiments. The strain-induced resistance changes for every CNT concentrations around the critical volume fraction were obtained by averaging more than 2,000 random Monte-Carlo simulations, which allows to obtain sufficient data for smoothing data scattering.

Figure 6(a) shows the conductance curves of the simulated domain according to the volume fraction for different maximum deviation angles of CNTs. Figure 6(b) also shows their percolation probability curves for each condition. As shown in the Fig. 6(a), it is observed that the conductance curve kept the slope until 20° of maximum deviation angles, but the slope of conductance curve was changed significantly after 30° of maximum deviation angle. Likewise, the percolation probability curves for each maximum deviation angle also show quite similar behavior for their critical volume fraction which shows significant changes after 30° of maximum deviation angle. For different maximum deviation angles, the critical volume fraction is around 3.0 vol% which is expected to produce sufficient resistance changes of fibre strain sensor as in the experiments.

The strain-induced resistance changes for different maximum deviation angles and volume fractions of CNTs in the polymer are also plotted in Fig. 6(c,d) with thousands of simulation trials. Comparing with Fig. 6(a,b), the strain-induced resistances change curves in Fig. 6(c) are changed monotonically as the maximum deviation angle of CNTs increases. Even though there are slight gap in between 20° and 30° of maximum deviation angles, it is thought that the curlier the CNTs are, more robust percolation network is formed, resulting in less change in the strain-induced resistance. From the results, it is expected that the curved CNT model provides a more realistic prediction of the electric characteristics of CNT/polymer composite as it includes the actual geometry of curly CNTs in the polymer composite.

Figure 6(d) shows the strain-induced resistance change curves for different volume fraction of every 0.4 vol% from 2.6 vol% to 4.2 vol% of CNTs. In this simulation, the maximum deviation angle Δ*θ*_*max*_ of curved CNTs was chosen to be 30° from Fig. 6(c) as an example of gentle CNT bending. The condition of Δ*θ*_*max*_ = 30° is thought to be acceptable value to model the curly CNTs observed experimentally and reported as SEM images in Fig. 5. As shown in Fig. 6(d), the strain-induced resistance curves are abruptly changed as the volume fraction of CNT decreases from 4.2 vol% to 2.6 vol%. The resistance change curves of sample #1 and #2 from our experiments are plotted together with dotted red and blue curves for the comparison with the simulation results.

Comparing the simulation results in Fig. 6(d) with the experimental results in Fig. 5(c), qualitative agreement was found in the resistance change curves between the simulation and experiment. From the simulation results, it is shown that the resistance change curve is abruptly changed, since the percolation network can be highly affected even by the small amount of the CNTs at around the critical volume fraction of the CNT/polymer composite. Here, the resistance change curves of the fabricated fiber strain sensor #1 and #2 are quite similar to the simulated resistance change curves for 2.6 vol% and 4.2 vol% CNT concentrations, respectively. From the comparisons between simulation and experiments, the effective volume fraction can be utilized to characterize the CNT dispersion of fabricated CNT/polymer composite. As a consequence, the effective volume fractions of the fabricate fibers #1 and #2 are presumed to be 2.6 vol% and 4.2 vol%, respectively. In summary our proposed simulation model describes the percolation phenomenon well and provides a framework for predicting the electromechanical characteristics of fiber strain sensors based on CNT/polymer composites.

## Conclusion

The electromechanical characteristics of the CNT/polymer composite used for a highly stretchable fiber strain sensor were analyzed by three-dimensional simulation of electrical percolation. The Monte-Carlo simulation method and a union-find algorithm were used to generate random CNT network and to investigate their percolation. A mathematical form for the rigid-body movement of the curved CNT for a large strain of a composite was developed. From the simulation, it was found that curvature of CNT significantly affects the electrical properties of a CNT/polymer composite. Two fiber strain sensors were fabricated from a CNT/polymer composite, their electromechanical properties are measured and compared with our simulation model. The strain-induced resistance curves obtained from our simulation model show good agreement with those of the fabricated fiber sensors. By comparing the simulation results with the experiment, the volume fraction of two fabricated fiber strain sensors were estimated to be 2.6 vol% and 4.2 vol%, respectively. In conclusion, our simulation model allows to obtain an accurate estimation of the electrical percolation phenomena affecting CNT/polymer composites which is suitable for curved CNTs under large strain condition and is useful for analyzing and predicting the electromechanical characteristics of highly stretchable fiber strain sensors based on such CNT/polymer composites.

## Data Availability

The source codes generated during the current study are available in the GitHub repository, https://github.com/smjung0/percolation3d. All data generated or analysed during this study are included in this published article.
